# Radiation-Sensitising Effects of Antennapedia Proteins (ANTP)-SmacN7 on Tumour Cells

**DOI:** 10.3390/ijms141224087

**Published:** 2013-12-11

**Authors:** Li Qing Du, Yan Wang, Chang Xu, Jia Cao, Qin Wang, Hui Zhao, Fei Yue Fan, Bing Wang, Takanori Katsube, Sai Jun Fan, Qiang Liu

**Affiliations:** 1 Institute of Radiation Medicine, Chinese Academy of Medical Sciences and Peking Union Medical College, Tianjin 300192, China; E-Mails: duliqing2004@126.com (L.Q.D.); bamboo201306@163.com (Y.W.); xuchang2001@yahoo.com (C.X.); jillpumc@gmail.com (J.C.); wangqin@irm-cams.ac.cn (Q.W.); fanfeiyue@irm-cams.ac.cn (F.Y.F.); fansaijun@gmail.com (S.J.F.); 2 Tianjin Key Laboratory of Molecular Nuclear Medicine, Tianjin 300192, China; 3 First Clinical Department of Medical Emergency Response Center for Nuclear Accidents, Ministry of Health, Tianjin 300192, China; 4 Tianjin Key Laboratory of Food and Biotechnology, Tianjin University of Commerce, Tianjin 300134, China; E-Mail: zhaohui@tjcu.edu.cn; 5 National Institute of Radiological Sciences, Chiba 263-8555, Japan; E-Mails: jp2813km@nirs.go.jp (B.W.); tkatsu@nirs.go.jp (T.K.)

**Keywords:** ANTP-smacN7 fusion protein, apoptosis, radiosensitivity

## Abstract

The objective of this study was to investigate the underlying mechanisms behind the radiation-sensitising effects of the antennapedia proteins (ANTP)-smacN7 fusion protein on tumour cells. ANTP-SmacN7 fusion proteins were synthesised, and the ability of this fusion protein to penetrate cells was observed. Effects of radiation on the expression of X-linked inhibitor of apoptosis protein (XIAP) were detected by western blotting. The radiation-sensitising effects of ANTP-SmacN7 fusion proteins were observed by a clonogenic assay. The effects of drugs and radiation on tumour cell apoptosis were determined using Annexin V/FITC double staining. Changes in caspase-8, caspase-9 and caspase-3 were detected by western blot before and after ANTP-SmacN7 inhibition of XIAP. The ANTP-SmacN7 fusion protein could enter and accumulate in cells; *in vitro* XIAP expression of radiation-induced tumour cells was negatively correlated with tumour radiosensitivity. The ANTP-SmacN7 fusion protein promoted tumour cell apoptosis through the activation of caspase3. ANTP-SmacN7 fusion protein may reduce tumour cell radioresistance by inducing caspase3 activation.

## 1. Introduction

In recent years, the rapid development of radio surgery provides a very effective method of treatment for cancer patients suitable for radiotherapy. However, the effects of radiotherapy on different tumours, either from different patients or with different pathologies, or even the same tumours, vary greatly. One important reason is the difference in the sensitivity of tumour cells to radiation. The abnormal regulation of apoptosis in tumour cells commonly affects the sensitivity of tumour cells to radiotherapy [1–3].

IAPs are a structurally related anti-apoptotic protein family, which includes XIAP, cIAP1, cIAP2, ML-IAP and Survivin. The IAP family of proteins inhibits apoptosis mainly by the binding of its BIR domain to caspase, which inhibits caspase activation and activity [4]. The increase in the expression of the IAP protein family and other apoptosis inhibitory proteins in tumour cells is the basis of tumour cell radiation resistance [5,6]. Therefore, targeting IAP molecules in tumour cells to enhance tumour cell sensitivity to drug therapy has become a hot research topic in radiobiology and oncology [7].

Smac/DIABLO is a bona fide mitochondrial protein with a typical amphipathic mitochondrial targeting sequence at its *N* terminus, which is presumably cleaved after mitochondrial entry [7]. During apoptosis, the mitochondrial Smac/DIABLO is released into the cytosol and binds to XIAP, which antagonises the interaction between XIAP and caspase 9, thereby promoting the activity of caspase 9, followed by caspase 3, and apoptosis. The *N* terminal peptide (AVPIAQK) of Smac/DIABLO (smacN7) can bind across a surface groove of the BIR 3 domain of XIAP in a mutually exclusive manner to caspase 9 [8–12]. Simones shows that the short synthetic peptides (smacN7) also promoted apoptosis [13]. Thus, the apoptosis inhibitory effect of IAP will be effectively inhibited if SmacN7 is introduced into cells. Complicating this process is the fact that SmacN7 molecules cannot enter from the extracellular space into cells alone [14]. Studies have shown that the third α-helix of the homeodomain of Drosophila antennapedia proteins (ANTP) (16 amino acid residues from 43 to 58) is the minimal peptide, which retains transduction functions, and this peptide can carry other proteins across the lipid bilayer and enter cells independent of receptors, channels, energy or endocytosis [1,15]. Thus, SmacN7 can be made to enter previously impenetrable tumour cells by generating a fusion protein by attaching the *C*-terminal lysine of SmacN7 to an *N*-terminal arginine of the 16 amino acid peptide with a proline spacer.

This project is based upon our previous work. Through cell and *in vivo* animal studies, ANTP-SmacN7 fusion proteins were introduced into tumour cells. After ionising radiation, the changes in cell survival, apoptosis, XIAP protein expression, Caspase-3, Caspase-8 and Caspase-9 activity were observed to determine the radiation-sensitising effects of ANTP-SmacN7 fusion proteins and the mechanism by which it promotes radiation-induced apoptosis.

## 2. Results

### 2.1. *In Vitro* Quantitative Analysis of XIAP Expression in Radiation-Induced Tumour Cells and Its Relationship with Tumour Radiation Tolerance

To observe the relationship between radiosensitivity of different tumour cells and XIAP expression, the EC109, NCL-H460, SGC7901 and HeLa tumour cell lines were irradiated with 8 Gy γ rays. The radiation tolerance of different tumour cells was determined by a clonogenic assay. XIAP expression across different tumour cells was detected by western blotting during the same period.

**Figure 1.**
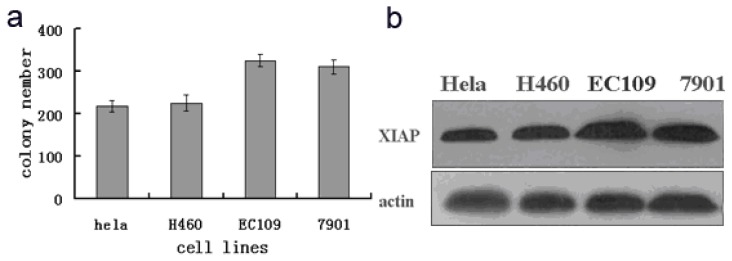
Relationship between XIAP expression of radiation-induced tumour cells and tumour radiosensitivity (**a**) Colony number of tumour cells after exposure to γ-ray; and (**b**) Western blot of the HeLa, H460, EC109 and 7901 tumour cell lines probed with antibodies to XIAP and β-actin.

As shown in Figure 1, XIAP protein expression in EC109 and SGC7901 cells was significantly higher than in H460 and HeLa cells, and clonogenic assay results also showed that after 8 Gy γ irradiation, the colony-formation rates of EC109 and SGC7901 cells were 81% and 77%, respectively. These rates were significantly higher than the HeLa and H466 cell lines (55% and 57%, respectively), and XIAP expression and the colony-formation rate differences were positively correlated (*R* = 0.82, *p* < 0.001). These data show that the decrease in radiosensitivity of tumour cells was related to an increase in the expression of XIAP. Therefore, EC109 was selected for subsequent experiments.

### 2.2. *In Vitro* Cell Permeability Experiments of the ANTP-SmacN7 Fusion Protein

**Figure 2.**
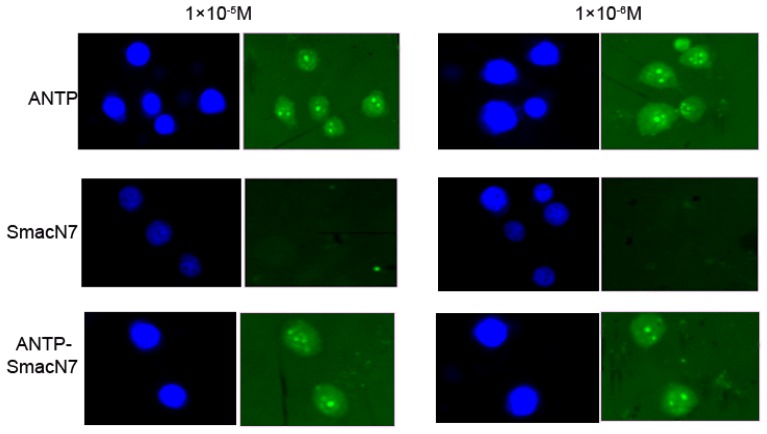
Comparison of cell transduction capabilities of SmacN7 and the ANTP-SmacN7 fusion protein. Blue shows nuclear staining (DAPI), green (FITC) shows carboxyl terminus-labelled ANTP, SmacN7 and ANTP-SmacN7.

To determine whether the ANTP-SmacN7 protein could enter tumour cells, the concentration of the polypeptide entering EC109 cells was measured by FITC fluorescence intensity, which corresponded to 1 × 10^−5^, 1 × 10^−6^ and 1 × 10^−7^ M of SmacN7, ANTP-SmacN7 and ANTP entering into EC109 cells, respectively. These results show that the ANTP-SmacN7 fusion protein can transduce and accumulate in cells, while SmacN7 alone cannot. Furthermore, this fusion protein is functional in EC109 cell at an effective initial concentration of 1 × 10^−6^ mol/L (Figure 2).

### 2.3. Evaluation of the Radiation Sensitising Effects of the ANTP-SmacN7 Fusion Protein

**Figure 3.**
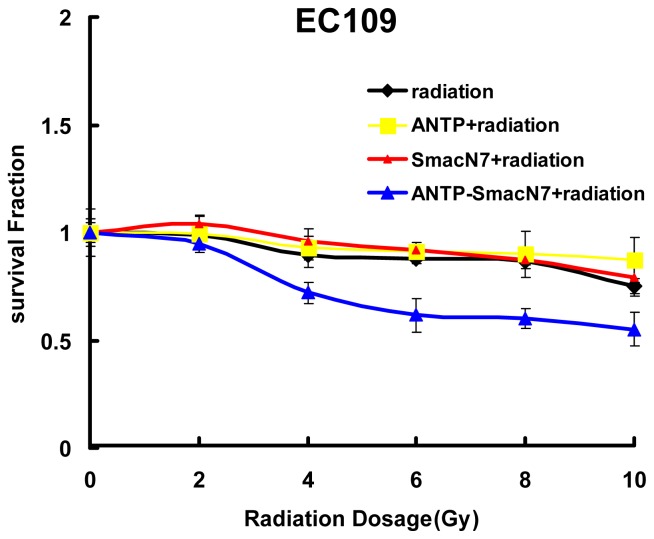
Radiation sensitising effects of ANTP-SmacN7.

Changes in cell survival among the three groups were evaluated by clonogenic assay, and the results are shown in Figure 3. The *D*_0_ values of the ANTP-SmacN7 combined radiation group and the pure irradiation group were 14.87 and 20.85, respectively; the SER was 1.41. These data show that the ANTP-SmacN7 fusion proteins had a radiation-sensitising effect on EC109 cells.

### 2.4. *In Vitro* and *in Vivo* Evaluation of the Effects of SmacN7 and ANTP-SmacN7 Fusion Proteins on Radiation-Induced Apoptosis

To observe the effects of ANTP-SmacN7 fusion proteins on radiation-induced apoptosis, 1 × 10^−5^ M ANTP, SmacN7 and ANTP-SmacN7 were incubated with EC109 cells, respectively. After 24 h, cells were irradiated with γ-rays at different doses (2, 4, 8, 10 Gy). Cells were harvested 48 h after irradiation. Apoptosis was detected using Annexin V and PI double staining; each experiment was repeated 3 times.

**Figure 4.**
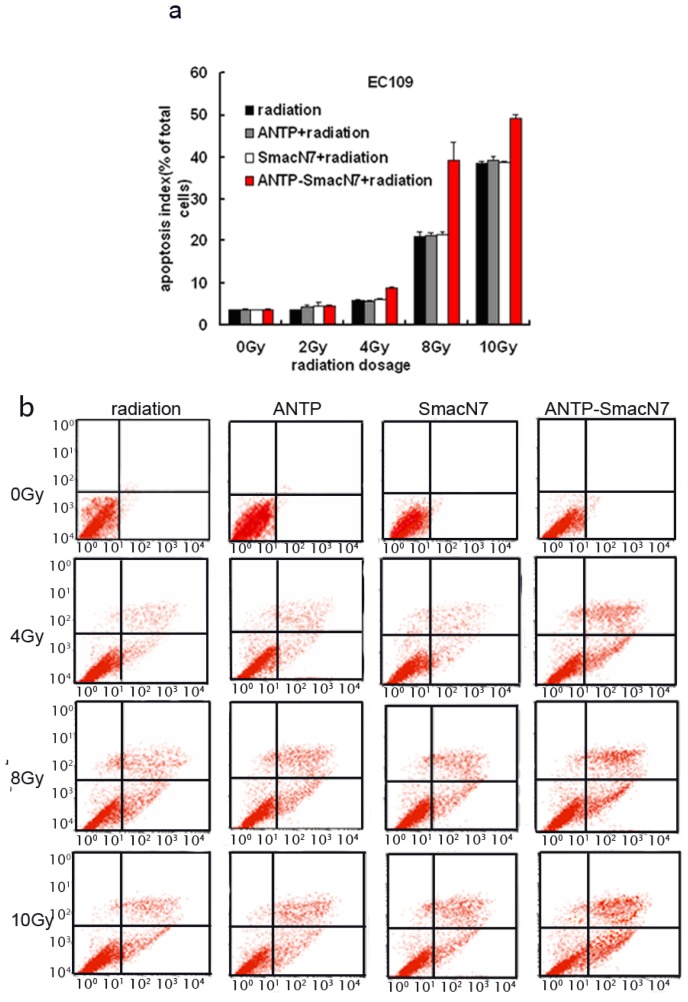
Effects of ANTP-SmacN7 on radiation-induced apoptosis (**a**). The apoptotic index of EC109 cells after treatment with ANTP, SmacN7, and ANTP-SmacN7 combined with radiation; and (**b**) Graph of the flow cytometry results.

The EC109 cell apoptosis rate increased gradually with irradiation dosage, and under 4, 8 and 10 Gy irradiation, the apoptotic rate of ANTP-SmacN7 group was increased significantly when compared to that of the ANTP, SmacN7 and pure irradiation groups (*p* < 0.01). The ANTP, SmacN7 and pure irradiation groups showed no significant differences in apoptosis rate (Figure 4; *p* > 0.05).

### 2.5. Effects of ANTP-SmacN7 Fusion Proteins on the Mitochondrial and Death Receptor Pathways of Tumour Cell Apoptosis

**Figure 5.**
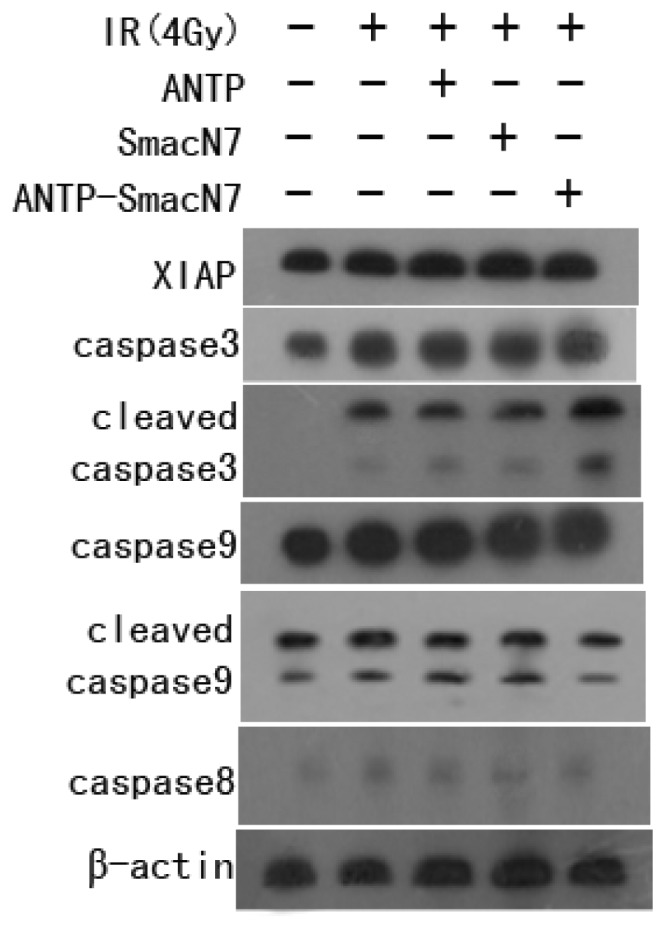
Effects of ANTP-SmacN7 on the tumour cell apoptotic pathway.

To observe the effects of XIAP apoptosis inhibition, SmacN7 or ANTP-SmacN7 was incubated with human oesophageal cancer EC109 cells. After 24 h, cells were irradiated with 4 Gy γ-rays. Cells were harvested 48 h after irradiation. Changes in caspase-8, caspase-9 and caspase-3 were detected by western blot before and after ANTP-SmacN7 inhibition of XIAP. The results show that radiation increased caspase-3 expression, while not affecting caspase-8, caspase-9 or cleaved caspase-9 (Figure 5). This result indicates that radiation-induced apoptosis might be principally mediated by caspase-3. While ANTP-SmacN7 combined with radiation did not promote further increases in caspase-3 protein expression, it increased the levels of cleaved caspase-3, indicating that ANTP-SmacN7 might promote the activation of the caspase-3 protein. No effects of ANTP-SmacN7 on the expression and activation of caspase-8 and caspase-9 proteins were found in this study.

## 3. Discussion

This study first defines the *in vitro* quantitative analysis of XIAP expression in radiation-induced tumour cells and its relation with the radiation tolerance of tumours and finds that tumour cells with a strong radiation resistance have relatively high XIAP expression. XIAP protein expression in EC109 cells was significantly higher than in 7901 or HeLa cells, and the clonogenic assay results also suggest that the colony-formation rate of EC109 cells after irradiation is higher than that of SGC7901 cells. Furthermore, XIAP protein expression is positively correlated with cell viability after irradiation, indicating that the decrease in the radiosensitivity of tumour cells is related to an increase in XIAP expression.

We then generated an ANTP-SmacN7 fusion protein by connecting the SmacN7 reactive group to a leader peptide of ANTP to promote SmacN7 cellular entry. Once in the cells, SmacN7 effectively blocks the apoptosis-inhibitory functions of IAP proteins, promoting a radiation-sensitising effect. The results from the clonogenic assay show that with increased radiation dosage, the survival rate of EC109 cells in the ANTP-SmacN7 treatment group is significantly lower than that in the ANTP, SmacN7 and pure radiation treatment groups. Apoptosis in EC109 cells increases gradually under 4, 8 and 10 Gy radiation, and the apoptosis rate in the ANTP-SmacN7 group is significantly higher than in the ANTP, SmacN7 and pure irradiation groups, which show no significant differences in the rates of apoptosis. These results indicate that ANTP can successfully transduce SmacN7 in tumour cells and that the ANTP-SmacN7 fusion protein plays a pro-apoptotic role in cells.

There are two main apoptosis signalling pathways. The first of these is the death receptor-mediated apoptosis signalling pathway. In this pathway, the intracellular apoptotic execution molecule caspase is activated by extracellular death receptors binding with corresponding ligands. The second pathway is the internal mitochondria-mediated apoptosis pathway, in which caspase is activated by mitochondrial release of cytochrome C. The results of the study show that radiation can increase caspase3 expression while not affecting caspase8, caspase9 or cleaved caspase9, suggesting that radiation-induced apoptosis may be mediated primarily through caspase3. Although ANTP-SmacN7 does not affect caspase3 protein expression, it can increase the number of cleaved caspase3 molecules, indicating that ANTP-SmacN7 may promote caspase3 protein activation. This activation occurs through the binding of ANTP-SmacN7 and XIAP, which blocks XIAP apoptosis inhibition by indirectly promoting caspase3 activation. In addition, no effects of ANTP-SmacN7 on caspase8 were found, indicating ANTP-SmacN7-mediated tumour cell apoptosis is not caspase8-dependent. These findings have been supported by the results from related studies.

## 4. Materials and Methods

### 4.1. Cell Culture

The EC109 oesophageal cancer, SGC7901 gastric cancer, HeLa cervical carcinoma and the NCL-H460 non-small cell lung human cancer cell lines were all stored in our laboratory. They were all normally cultured in RPMI 1640 medium (Gibco, Carlsbad, CA, USA) containing 10% FBS at 37 °C in 5% CO_2_.

### 4.2. ANTP-SmacN7 Fusion Protein

Cell-penetrating functions of the ANTP-SmacN7 fusion protein were observed by *C*-terminal FITC labelling. The SmacN7, ANTP-SmacN7 and ANTP recombinant proteins were synthesised by Shanghai Sangon Biotech Company and contained the following amino acid sequences:

ANTP: Arg-Gln-Ile-Lys-Ile-Trp-Phe-Gln-Asn-Arg-Arg-Met-Lys-Trp-Lys-Lys-FITC;
SmacN7: Ala-Val-Pro-Ile-Ala-Gln-Lys-Pro-FITC;
ANTP-SmacN7: Ala-Val-Pro-Ile-Ala-Gln-Lys-Pro-Arg-Gln-Ile-Lys-Ile-Trp-Phe-Gln-Asn-Arg-Arg-Met-Lys-Trp-Lys-Lys-FITC.

### 4.3. *In Vitro* Permeability Experiment of ANTP-Smac N7 Fusion Proteins

The medium from nominally growing tumour cells was aspirated. Cells were washed twice with ice-cold PBS and collected by conventional digestion. Cell suspension was prepared using RPMI1640 medium containing 10% FBS. The cell concentration was adjusted to 1 × 10^5^ cells/mL. A total of 200 μL of cell suspension was added to slides placed in a six-well plate; 24 h after cell adherence, the medium was discarded, and corresponding concentrations of target drugs were added. Cells were cultured for 3 h and 5 mg/L DAPI in 50 μL was added for mounting. Cellular drug uptake was observed under an upright fluorescence microscope (Olympus BX61, Tokyo, Japan).

### 4.4. Western Blot

Subconfluent cells were collected and were pyrolysed using MER-Pierce lysate. Protein quantification was conducted according to BCA Protein Assay Kit instructions (Takara, Shiga, Japan). A total of 50 μg of proteins were taken for discontinuous polyacrylamide gel electrophoresis (SDS-PAGE), electrotransferred to a polyvinylidene difluoride membrane (PVDF) and blocked with TBST containing 5% skim milk at 4 °C overnight. The membranes were then incubated with primary antibodies at room temperature for 2 h; the membranes were then washed, incubated with a corresponding horseradish peroxidase-conjugated secondary antibody at room temperature for 1 h and detected by ECL chemiluminescence. β-Actin was used as an internal control.

Primary antibodies included XIAP (abcam company No.: ab21278, 1:300 dilution); caspase-8 (abcam company No.: ab32125, 1:3000 dilution); caspase-9 (abcam company No.: ab32539, 1:3000 dilution); caspase-3 (abcam company No.: ab32351, 1:5000 dilution) and actin (abcam company No.: ab3280, 1:3000 dilution).

### 4.5. Clonogenic Assay

Each well of a 6-well plate was seeded with 400 cells for 24 h, followed by ANTP-SmacN7 addition for 24 h. The cells were subsequently irradiated in air at room temperature by a ^137^Cs γ source (Atomic Energy of Canada Limited, Chalk River, ON, Canada, CAMMA-CELL40). The source target distance was 30 cm, and the dose rate was 0.793 Gy/min. After irradiation, cell cultures were grown at 37 °C and terminated after 10 days. Cells were fixed and stained, and the experiment was repeated twice. At least 50 cells were defined as a clone colony. Clone-formation rate (%) equalled the number of clone colony/number of inoculated cells ×100%. A cell survival curve was fitted based on a single-hit multi-target equation, and the curve parameter *D*_0_ value was calculated. The radiosensitisation ratio (SER) equalled the *D*_0_ value of irradiation group/*D*_0_ value of dosed irradiation group.

### 4.6. Apoptosis Detected Using Annexin V-PITC/PI Double Staining Method

Cells were trypsinised with EDTA-free trypsin and washed twice with PBS (centrifuged at 2000 rpm for 5 min). A total of 5 × 10^5^ cells were collected and suspended in 500 μL of binding buffer with 5 μL Annexin V-FITC (Sigma-Aldrich, St. Louis, MO, USA); the suspension was mixed evenly, and 5 μL of propidium iodide was added and mixed evenly. The cell suspension was protected from light and incubated at room temperature for 5–15 min. Fluorescence was detected by flow cytometry (BD Biosciences, San Jose, CA, USA) within 1 h (excitation wavelength *E_x_* = 488 nm; emission wavelength *E_m_* = 530 nm). Green fluorescence of Annexin V-FITC was detected by FITC channel (FL1); PI red fluorescence was detected by PI channel (FL2 or FL3).

### 4.7. Statistical Analysis

The cell survival curves were fitted by a single-hit multi-target equation. Analysis was conducted using SPSS13.0 statistical software (SAS Institute Inc., Cary, NC, USA). Differences among groups were analysed using two-way analysis of variance. Pairwise comparisons were conducted using Dunnett’s *t*-test. A cutoff of *p* < 0.05 was taken for statistical significance.

## 5. Conclusions

In summary, the increase in the expression of the IAP protein family and other apoptosis inhibitory proteins in tumour cells is the basis by which tumour cells generate radiation resistance. Therefore, IAP molecules in tumour cells represent a target, which, when inhibited, enhances tumour cell sensitivity to treatment. IAP molecules have become a hot research topic in radiobiology and oncology therapeutics [11,16], and this study further defines the relationship between the expression of the IAPs and cell radiosensitivity.

Tumour tolerance to radiation during radiotherapy has been puzzling. We show that the ANTP-SmacN7 fusion protein can specifically block the apoptosis inhibitory effect of XIAPs proteins, thereby reducing the radioresistance of tumour cells. Thus, once a therapeutic based on the ANTP-SmacN7 fusion protein enters the clinic, it will greatly improve the efficacy of tumour radiotherapy, prolong the survival of tumour patients and improve their quality of life, which will ultimately generate an enormous social and economic benefit.
